# Cell-specific transcriptional signatures of vascular cells in Alzheimer’s disease: perspectives, pathways, and therapeutic directions

**DOI:** 10.1186/s13024-025-00798-0

**Published:** 2025-01-29

**Authors:** Soumilee Chaudhuri, Minyoung Cho, Julia C. Stumpff, Paula J. Bice, Özkan İş, Nilüfer Ertekin-Taner, Andrew J. Saykin, Kwangsik Nho

**Affiliations:** 1 Department of Radiology and Imaging Sciences, Center for Neuroimaging, Indiana University School of Medicine, Indianapolis, IN USA; 2 Indiana Alzheimer’s Disease Research Center, Indiana University School of Medicine, Indianapolis, IN USA; 3 Medical Neuroscience Graduate Program, Stark Neurosciences Research Institute, Indiana University School of Medicine, Indianapolis, IN USA; 4 Department of Digital Health, Samsung Advanced Institute for Health Sciences & Technology (SAIHST), Samsung Medical Center, Sungkyunkwan University, Seoul, Republic of Korea; 5 Ruth Lilly Medical Library, Indiana University School of Medicine, Indianapolis, IN USA; 6 Department of Medical and Molecular Genetics, Indiana University School of Medicine, Indianapolis, IN USA; 7 Center for Computational Biology and Bioinformatics, Indiana University School of Medicine, Indianapolis, IN USA; 8 Department of Neuroscience, Mayo Clinic, Jacksonville, FL USA; 9 Department of Neurology, Mayo Clinic, Jacksonville, FL USA

**Keywords:** Alzheimer’s disease (AD), Single cell or single nuclei transcriptomics (scRNAseq or snRNAseq), Cerebrovascular cells, Neurovascular unit (NVU), Endothelial cells, Smooth muscle cells, Fibroblasts, Pericytes

## Abstract

Alzheimer’s disease (AD) is a debilitating neurodegenerative disease that is marked by profound neurovascular dysfunction and significant cell-specific alterations in the brain vasculature. Recent advances in high throughput single-cell transcriptomics technology have enabled the study of the human brain vasculature at an unprecedented depth. Additionally, the understudied niche of cerebrovascular cells, such as endothelial and mural cells, and their subtypes have been scrutinized for understanding cellular and transcriptional heterogeneity in AD. Here, we provide an overview of rich transcriptional signatures derived from recent single-cell and single-nucleus transcriptomic studies of human brain vascular cells and their implications for targeted therapy for AD. We conducted an in-depth literature search using Medline and Covidence to identify pertinent AD studies that utilized single-cell technologies in human post-mortem brain tissue by focusing on understanding the transcriptional differences in cerebrovascular cell types and subtypes in AD and cognitively normal older adults. We also discuss impaired cellular crosstalk between vascular cells and neuroglial units, as well as astrocytes in AD. Additionally, we contextualize the findings from single-cell studies of distinct endothelial cells, smooth muscle cells, fibroblasts, and pericytes in the human AD brain and highlight pathways for potential therapeutic interventions as a concerted multi-omic effort with spatial transcriptomics technology, neuroimaging, and neuropathology. Overall, we provide a detailed account of the vascular cell-specific transcriptional signatures in AD and their crucial cellular crosstalk with the neuroglial unit.

**Graphical Abstract:**

Endothelial and mural cell types mediate dysregulated transcriptional pathways and cell-cell interactions in AD. The neurovascular unit (NVU) is composed of various cell types, including endothelial cells, mural cells (pericytes, smooth muscle cells), fibroblast neurons, microglia, and astrocytes. Dysregulated transcriptional pathways in AD involve multiple pathways, notably immune responses, and angiogenesis common to both endothelial and mural cells. Additionally, pathways involving neuroinflammation and amyloid clearance are prominent in endothelial cell types, while mural cells exhibit pathways related to growth factors, cytoskeletal remodeling and synaptic function. In addition, crosstalk within the NVU and gliovascular unit (GVU) is altered in AD, with altered cell-cell communication evident, with increased interactions between endothelial cells, pericytes, neurons, and microglia, and decreased interactions between endothelial cells, fibroblasts, astrocytes, and neurons. Figure created with BioRender.com. Abbreviations: AD, Alzheimer's disease; NVU, Neurovascular unit; CNS, Central Nervous System.

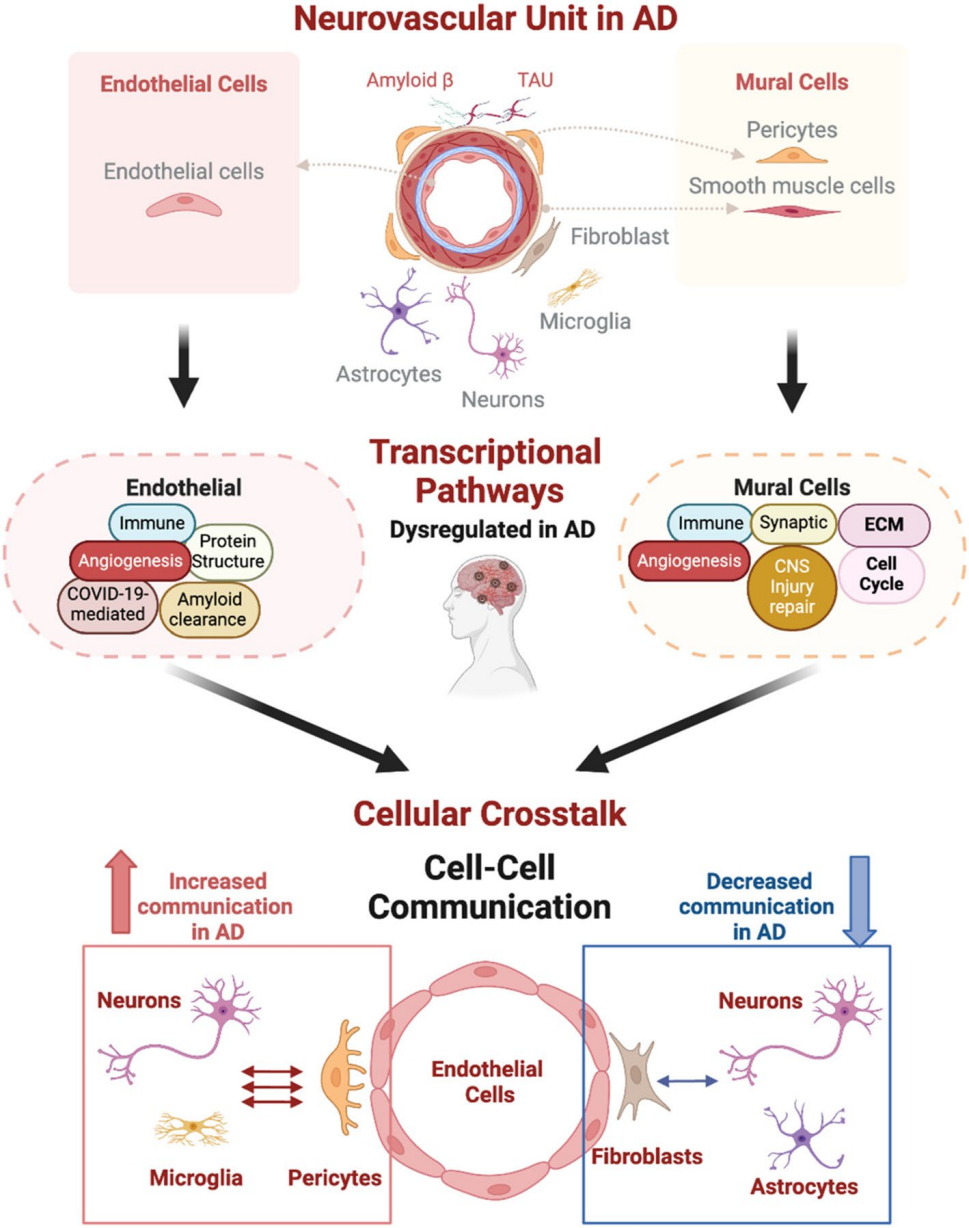

**Supplementary Information:**

The online version contains supplementary material available at 10.1186/s13024-025-00798-0.

## Background

### Dysfunction of neurovascular unit (NVU) and gliovascular unit (GVU) in Alzheimer’s disease

Alzheimer’s disease (AD) is a devastating neurodegenerative disorder characterized by significant cognitive decline and memory loss. It has been estimated that 13 million Americans will be living with AD by 2050 [1, 2]. After decades of clinical trial failure associated with anti-amyloid immunotherapy treatments for AD, [3–7] the recent FDA approval of lecanemab and donanemab as disease modifying therapies mark a significant breakthrough in the field [8]. However, these drugs are not a cure for AD and have been reported to have multiple adverse side effects including brain swelling and brain bleeding [9]. These findings highlight the lack of a safe therapy for AD, underscore the complex nature of AD pathogenesis, and necessitate integrated approaches targeting multiple realms of AD dysfunction. Of note—is the remarkable growth in the field attributing cerebrovascular dysfunction as an additive (co-pathology) or synergistic contributor to AD [10, 11]. Compelling evidence from various neuroimaging studies, including several conducted by our research group, has implicated involvement of various cerebrovascular factors in the neurodegenerative processes known to impact AD, including compromised blood–brain barrier, altered cerebral blood flow, increased cerebrovascular resistance, and impaired vascular responses [12–17]. These factors often arise from and/or exacerbate the dysfunction of the neurovascular unit (NVU), ultimately leading to AD pathology. The NVU comprises structural and functional interaction among neurons and cells surrounding the vasculature such as endothelial cells, fibroblasts, pericytes, and astrocytes, whereas GVU represents cerebrovascular cells and glia which constitute the blood–brain barrier (BBB) [18, 19]. Disruption of the coordinated functioning of these components has been linked to AD pathogenesis and BBB dysfunction in AD [20–23]. Furthermore, these neurovascular and gliovascular interactions characterize brain pathologies. Amyloid-beta, a pathological hallmark of AD, has been linked with disruption of cerebral circulation by targeting NVU vascular and perivascular cells [24–32].

Overall, recent discoveries on the cellular mechanisms and pathways associated with neurodegeneration in AD, including those from our group [33–35], have provided insight into the specific contribution of vascular cell types to mechanisms contributing to AD.

### Understanding human brain vasculature in AD using single-cell transcriptomics

Recent progress in the application of single-cell and single-nucleus technologies in AD patients has enriched our understanding of cell-type-specific contributions to AD [33, 36–50]. Some of these have contributed to significant advances in recognizing molecular alterations in major brain and blood cell types such as neurons, microglia, astrocytes, and oligodendrocytes, and how they can be leveraged for cell-specific targeted therapy for AD [51–62]. However, knowledge of the diverse human cerebrovascular cells was limited due to their sparsity and dispersion in the brain tissue, until recent studies made significant strides in dissecting the transcriptional diversity in lesser-known cell types such as brain endothelial cells (BECs), smooth muscle cells (SMCs), fibroblasts and pericytes, by adapting new bench techniques and computational pipelines to isolate vascular cells from post-mortem human brains [36, 63, 64]. Additionally, significant developments have elucidated how changes in vascular cell subpopulation and transcription factors, which affect the regulatory networks of these cells, may be important in identifying precise cellular targets for therapeutic developments in AD. Furthermore, notable advancements have been achieved in understanding the alterations occurring in vascular cell subpopulations and transcription factors that impact the regulatory networks of these cells [63–65]. These findings hold great significance in pinpointing specific cellular targets for the advancement of therapeutic interventions in AD. It has been established that gaining knowledge about cerebrovascular cells at the single-cell level and understanding their regulatory features can provide valuable insights into comprehending the integrity of the blood–brain barrier and developing therapeutics for AD.

Here, we highlight important findings from AD studies leveraging single-cell transcriptomics from post-mortem human brain tissue, expanding the crucial repository of single-cell reviews [37–39, 44, 53] and advancing knowledge by emphasizing the cell-type specific transcriptomic contribution of vascular cells to AD. We focus on publications that leverage single-cell transcriptomics, including single-cell RNA-sequencing (scRNA-Seq) and single-nucleus RNA-sequencing (snRNA-Seq), to uncover the detailed transcriptional underpinnings of brain vascular cells, their interactions with the neuroglial units and astrocytes, and the implications for AD therapeutics. There has been limited investigations that consolidate the transcriptional signatures of vascular cells with a direct focus on AD; therefore, to our knowledge, this is the first review of the transcriptional landscape of vascular cells of the NVU in AD human studies.

## Methodology

**Fig. 1:**
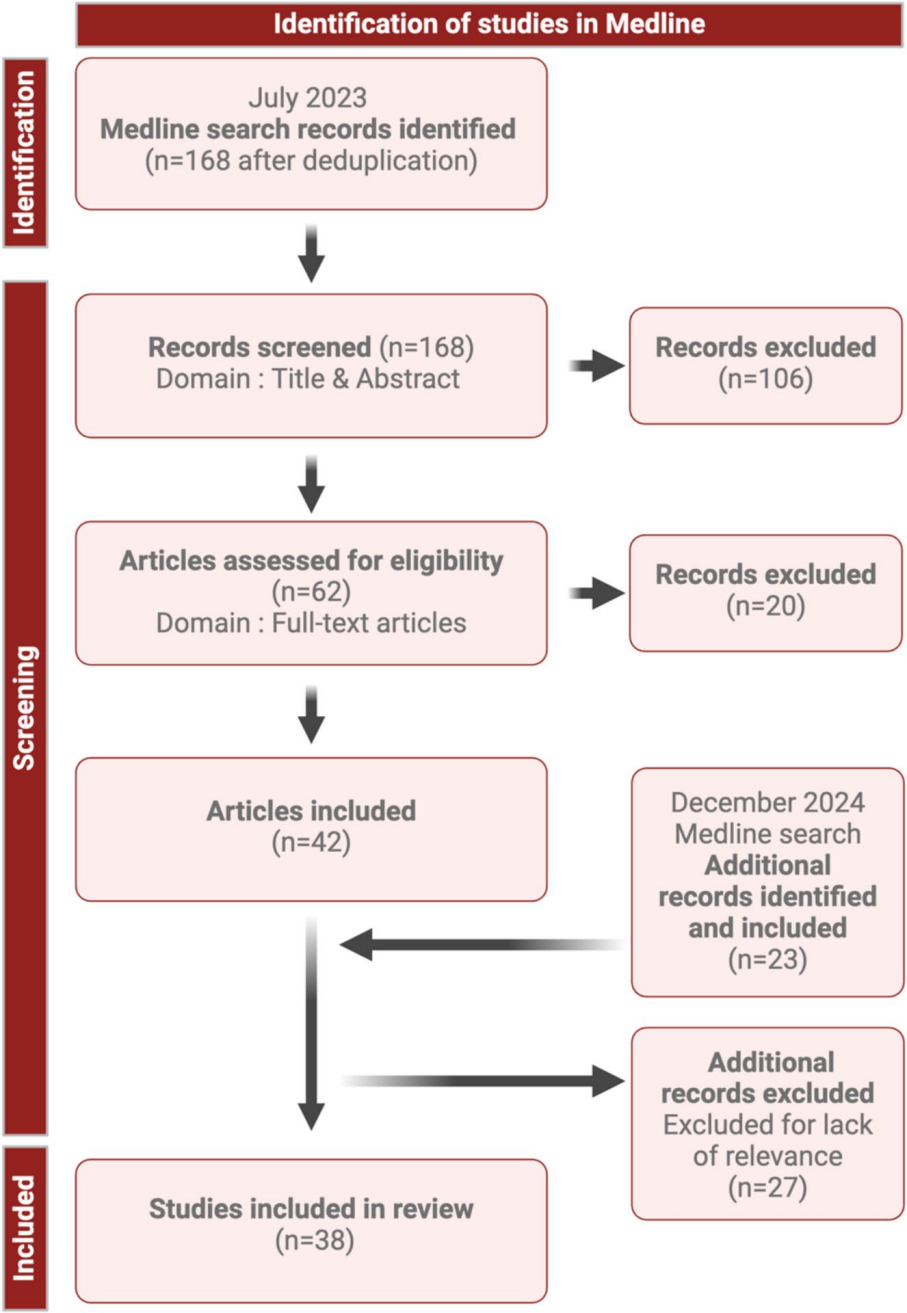
Schematic flowchart depicting methodology for this narrative review including literature search, screening, and inclusion. Abbreviations: AD, Alzheimer’s disease; ADRD, Alzheimer’s disease and related dementias; CAA, Cerebral Amyloid Angiopathy

A comprehensive literature search on PubMed with MeSH terms including “vascular cells,” “neurovascular unit, “Blood–Brain Barrier,” “Alzheimer’s Disease,” and “single cell transcriptomics” was conducted on September 24. 2023 (Fig. 1). Clear inclusion and exclusion criteria were established a priori to ensure that pertinent publications were selected that encompass human studies in AD. A medical librarian (J.S) composed and conducted comprehensive search strategies in MEDLINE (Ovid), Embase (Ovid), and the Cochrane Database of Systematic Reviews, identifying 168 papers. The systematic review management tool Covidence removed duplicate records, and the research team screened in two stages (S.C and M.C), first combining title and abstract screening and then reviewing the full-text. At each stage of the screening process, two reviewers independently screened each article, and conflicts were resolved by consensus. We focused solely on literature employing single-cell methodologies and datasets in the primary workflow of the research study. As part of this process, all articles were manually reviewed to ensure that the resulting studies leveraged single-nuclei methodology or single-cell data in brain or brain-related tissues of Alzheimer’s patients. After a full-text review, the research team identified 38 articles that met this updated inclusion criteria. Data from these articles were extracted using Covidence and included in this review (Supplementary Table 1).

There are inherent limitations to using a meta-analytic approach to summarize findings from single-cell transcriptomic studies. Some of the common key challenges arising from diverse methodological considerations include variations in sampling strategies, differences in tissue processing, cell type isolation, and study populations, as well as disparities in sequencing technologies, computational pipelines, and data normalization methods. These can often complicate cross-study comparisons, making it challenging to interpret cell-specific biological findings from complex transcriptomics output. To clarify some of these issues, we have detailed the technical aspects and unique methodological approaches employed in each summarized study, outlined in the supplementary tables. We hope this transparent presentation emphasizes the importance of methodological rigor and serves as a resource for future efforts to harmonize single-cell transcriptomics studies in complex biological questions.

## Cellular and subcellular diversity of the neurovascular unit in humans

The neurovascular unit in humans comprises various cell types, with vascular cells constituting a minority. Advances in single-cell and single-nucleus technologies, along with in-silico enrichment pipelines [57, 65] and ex-vivo extraction protocols, such as Vessel Isolation and Nuclei Extraction for Sequencing (VINE-Seq) [63] and the Blood Vessel Enrichment (BVE) protocol [64], have improved the detection of rare vascular cell types, revealing previously uncovered diversity of endothelial and mural cells throughout human development, neurogenesis, and to some extent, in neurodegeneration [63–67]. A previous review from our research group [68] provides a comprehensive overview of the function of the various NVU cells and their progenitors involved in adult neurogenesis, their dysfunction in neurodegenerative disease, and the potential for therapeutic targeting of neurogenesis pathways in diseases like AD, Schizophrenia, Huntington’s disease, etc. However, limited but significant recent studies have focused on the distinct vascular cell types within the NVU/GVU and their roles in neurodegeneration associated with AD. These studies have made notable progress in unraveling the intricate cellular and subcellular diversity of NVU/GVU cells, bringing us to a more comprehensive understanding.

Recent studies utilizing snRNA-Seq to investigate AD pathology have determined specifically that the most abundant of the most prevalent vascular cells, brain endothelial cells (BECs), are capillary in nature, with subsets of arterial and venous endothelial cells (Supplementary Table 2) [63, 64]. Another subtype of BEC, termed mitotic endothelial cells, is exclusively detected in the prenatal human brain and is absent in the adult brain [66]. BECs exhibit distinguished characteristics from peripheral endothelial cells that help them contribute to blood–brain barrier (BBB) formation [69, 70]. Among other vascular cells, single-cell studies have classified smooth muscle cells (SMC) into two subtypes, arterial (aSMC) and venous (vSMC), as seen in several experimental models that study SMC differentiation in the context of health and diseases [71, 72]. Pericytes have been grouped into transport pericytes (T-pericytes) and matrix pericytes (M-pericytes). T-pericytes have been found to be enriched in small-molecule transmembrane transporters such as the GABA transporter SLC6A1 and the glutamate transporter SLC1A3, whereas the matrix pericytes (M-pericytes) are enriched with extracellular matrix (ECM) organization [73, 74]. We recently identified a pericyte cluster from human snRNA-Seq data from AD and control donors, enriched for solute transport and ECM organization [33]. We found that pericytes were clearly delineated from other vascular cell types, such as endothelia and perivascular fibroblasts and exhibited the largest number of differentially expressed genes (DEGs) among the vascular clusters, indicating their selective vulnerability in AD. Studies of fibroblasts [63–65, 75] in the human brain have also shown their existence in two distinct clusters: perivascular fibroblasts expressing ECM proteins and meningeal fibroblasts expressing solute transporters. Research in animal models [76] has highlighted the importance of these perivascular fibroblasts in the brain vasculature, but their limited number has constrained their transcriptional investigation using snRNA-Seq analysis in humans. However, a recent AD study [65] detected perivascular fibroblasts in post-mortem human brains, uncovering three distinct subtypes of perivascular fibroblasts (Type I, Type II, and Type III) with different gradients of gene expression. All subtypes expressed specific gene sets, and pathway analysis revealed distinct functional roles for each subtype, as previously described in the literature [75]. Multi-regional single-cell transcriptomics in these studies has also shown that there are significantly higher proportions of fibroblasts than pericytes and endothelial cells in the entorhinal cortex, hippocampus, and thalamus. The differential distribution of these cell types in the entorhinal cortex, hippocampus, and thalamus suggests region-specific roles and contributions to the neurovascular unit. Given the known functions of fibroblasts, pericytes, and endothelial cells in regulating vascular integrity, blood–brain barrier function, and neurovascular coupling, the observed variations in their proportions from recent single-cell studies may suggest regional susceptibility to vascular dysfunction and neurodegenerative processes.

However, little is known about the cellular and transcriptional diversity of two understudied components of the human brain vasculature: ependymal cells and perivascular macrophages. Although a compromised ependymal barrier function plays a role in neurodegenerative disease [77, 78] and perivascular macrophages are involved in brain amyloid collection and the clearance in AD [79–81], previous snRNA-Seq studies conducted on human brains could not fully characterize the extensive diversity of these cells at a single-cell resolution.

## Transcriptional landscape of human vascular cells in AD: relationship to biological pathways

### Brain endothelial cells

As primary constituents of the neurovascular system, BECs play a pivotal role in maintaining BBB integrity. Although the exact mechanisms of endothelial cell dysfunction leading to BBB abnormalities in AD remains under scrutiny, findings from several recent single-cell studies [33, 63–65, 82–85] showed that there was a notable increase in the angiogenic state in the endothelial cells from AD patients. Specifically, these cells showed increased expression of angiogenic factors and receptors like CLDN5 (Claudin-5, tight junction protein, involved in the maintenance of BBB permeability and integrity) [86], ERG (transcription factor implicated in angiogenesis and vascular development) [87]*,* FLT1 (receptor-1 for vascular endothelial growth factor) [88, 89], *and* VWF (Von Willebrand Factor, regulating hemostasis and thrombosis) [90]. This angiogenic state has also been shown to be intricately linked with the altered presentation of antigen-presenting machinery (*B2M* and *HLA-E*) and distinct transcriptomic changes in different BEC subpopulations [82]. Out of the 7 identified transcriptionally distinct BEC subpopulations, only three contributed to major transcriptional changes in AD and had differentially expressed genes (DEGs) that were upregulated in AD and corresponded to major biological functions, including angiogenesis, transmembrane transport, antigen presentation, metal ion homeostasis, cellular respiration, and rRNA processing. It was also found that transcriptional regulators of these DEGs were linked to immune response; this finding was corroborated by a recent study that used a fluorescence-activated cell sorting (FACS)-based RNA-Seq approach to observe that endothelial cells from AD human brains had a downregulated expression of *CR1*, a gene that encodes for a receptor extremely crucial in immune clearance and surveillance [66]. Another study that relied on a unique vessel extraction and enrichment protocol to look at both the hippocampus as well as the cortex found that hippocampal endothelial cells preferentially showed higher levels of interferon-γ (IFNγ*)* signaling and accompanied hippocampal pericyte loss, providing a model for hippocampal vascular dysfunction [63, 64, 91–93]. In addition to being highly enriched in CTNNB1 (Catenin Beta 1), a key player in maintaining BBB integrity through cell adhesion pathways, a recent study found that BEC transcriptomes from five different cortical regions were upregulated in protein folding genes and had a distinct response to amyloid pathology regardless of brain region [94]. Interestingly, this study also reported that ABCB1 (P-glycoprotein), which is involved in amyloid-beta clearance at the neurovascular unit, was specifically downregulated in capillary endothelial cells from AD brains [95–97]. A few studies have also reported insulin-signaling genes to be dysregulated in BEC, with different cell subpopulations expressing differential levels of genes corresponding to the presence of insulin receptors in these clusters.

Most studies have reported no sex-specific stratification of gene expression in these endothelial cells. However, a study [98] focusing on the mid-frontal cortex of AD patients with *TREM2* mutations found that pericytes/endothelial cells from male donors had higher DEGs. In specific studies, BECs contained the most AD-related GWAS genes, and most of these genes were enriched in protein endocytosis and transcytosis processes [63–65].

**Fig. 2:**
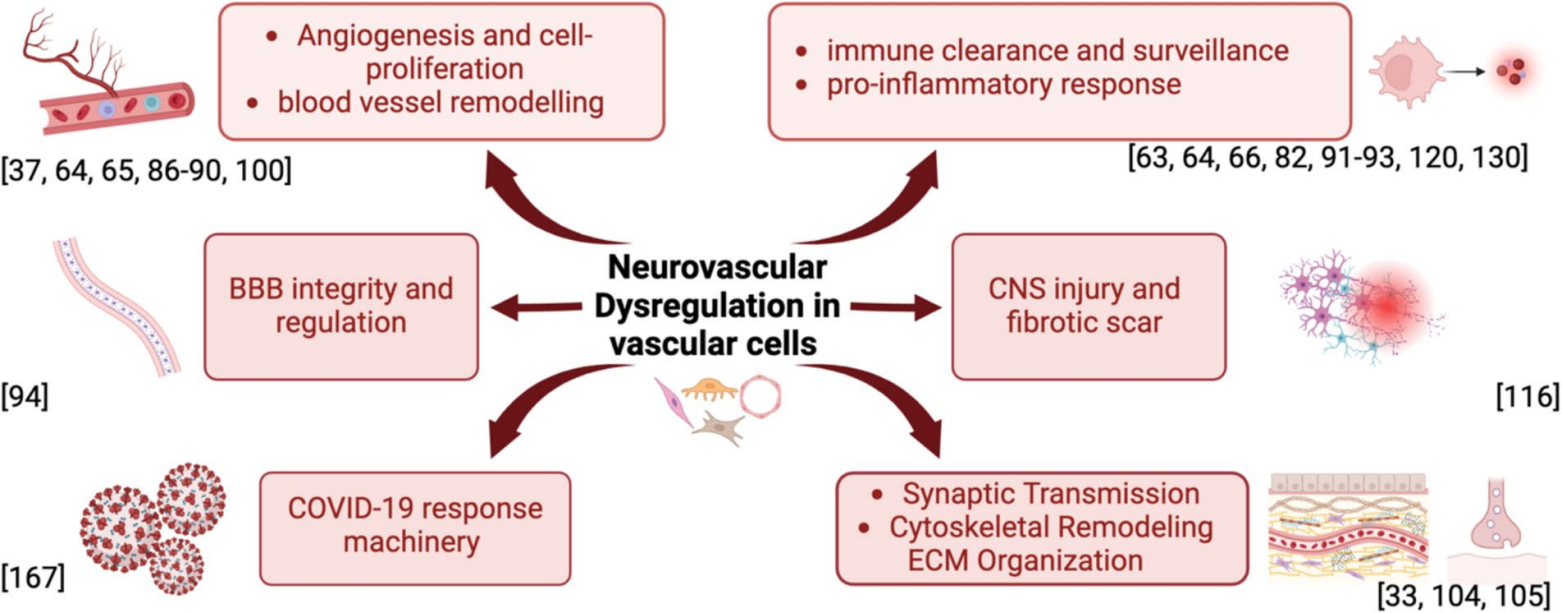
Dysregulated pathways identified by single cell RNA analysis of the human cerebrovascular cells. Red and blue arrows denote upregulated and downregulated transcriptomic pathways in AD, respectively. Angiogenesis and immune response are strongly associated and upregulated in endothelial and mural cells, whereas machinery supporting synaptic transmission, cytoskeletal remodeling, and BBB regulation show possible downregulation in response to AD. Figure created with BioRender.com. Abbreviations: NVU, Neurovascular unit; BEC, brain endothelial cells; CNS, Central Nervous System; BBB, Blood–brain barrier

Studies that relied on bioinformatics analysis and validation of multiple single-cell gene expression datasets from post-mortem brain tissue and age and gender-matched controls, like Soreq et al. [99], also validated some of the aforementioned findings. The study indicated that the transcriptomic profiles of endothelial cell clusters exhibited increased expression of ABCB1 and EBF1[99]. Furthermore, the proportion of endothelial cells was found to be higher in the AD samples compared to neurological control samples in the same study. Overall, most of these data-driven studies of vascular cells showed that cell-type specific transcriptomic changes in AD were associated with four major molecular pathways, with two of them (angiogenesis and immune response) being strongly associated with brain endothelial cell function (Fig. 2).

In the central nervous system (CNS), progesterone has been demonstrated to exert a neuroprotective effect through the hormone receptor PGRMC1 (Progesterone receptor membrane component 1), but it has also been established that *PGRMC1* binds to Aβ oligomers in AD brain tissue, mediating the possible synaptotoxicity effects of Aβ. This is relevant because a single-cell transcriptomics study, [100] which investigated the role of *PGRMC1* in endothelial cells in AD, found that expression levels of *PGRMC1* were low in the frontal and temporal cortex tissues of AD patients compared to controls. Still, the DEGs associated with *PGRMC1* were highly enriched in the regulation of cell proliferation and angiogenesis.

Transcriptional patterns in endothelial cells offer a nuanced perspective on the intricate dynamics within the NVU, shedding light on the physiological state of normal brains and the alterations occurring in AD. These single-cell transcriptomic studies have broadly implicated dysregulated angiogenesis, cell proliferation, and immune response pathways in BECs of AD patients (Fig. 2). These studies provide critical evidence that observed dysfunction of endothelial cells exacerbates the profound inflammatory and neurotoxic responses followed by astrogliosis at the cellular level in AD. Although most studies have shown no significant reduction in cell density and no region or sex-specific differences in relative abundance and gene expression patterns in AD human brains, a few studies have found region-specific and species-specific patterns of BEC density reduction [63, 101, 102]. Recent research has demonstrated that AD in human and murine brains is marked by widespread loss of vascular cells in general [103]. Therefore, more single-cell transcriptomic research is needed to fully understand the exact spatial characterization of BECs and their subtypes in the underlying pathogenesis of AD.

### Mural cells and fibroblasts

Studies have noted that mural cells account for the most transcriptional changes in AD [36, 63–65]. Of note—is the subtype M-pericytes, which is involved in ECM organization and has exhibited selective vulnerability for AD through snRNA-Seq in humans and immunostaining validations in mouse models. Like their endothelial cell counterparts, pericytes were also found to be enriched in *BACH1*, a gene that is responsible for encoding transcription factors for oxidative stress, inflammation, and angiogenesis. PDGFRβ (a platelet-derived growth factor β that plays a crucial role in cell proliferation, differentiation, and survival) was also downregulated in pericytes from AD. In pericytes, most downregulated DEGs were mapped to synaptic transmission and remodeling of cytoskeletal machinery, which is consistent with what we see in human and mouse AD studies [33, 104–107]. Immunohistochemical validations in post-mortem brain tissue have made similar conclusions, with such studies reporting a reduction in pericyte marker PDGFRβ, synaptic markers such as synaptophysin, and tight junction proteins such as CLDN5, occludin, and ZO-1 (zonula occludens) [108–113].

A single-cell RNA-Seq study was conducted on olfactory mucosal (OM) tissue from AD patients and aged-matched controls to understand transcriptomic differences in fibroblasts from the OM tissue [114]. After comparing the OM transcriptome with snRNA-Seq data of the entorhinal cortex from the same AD patients, this study found that both datasets had eight common DEGs between control and AD groups. This finding underscores the importance of dysregulated genes in AD regardless of the tissue type assessed and suggests that the entorhinal cortex and OM are both vulnerable to the pathogenesis of early AD and exhibit disease-specific cell-type alterations in fibroblast transcriptomes. One of the more recent studies [65] uncovered three distinct subtypes of perivascular fibroblasts and showed that transcriptomic profiles of type I fibroblasts corresponded with pathways regulating fibrosis during injury to CNS, whereas type II and type III fibroblast subtypes were enriched in pathways related to cell fate determination and growth factors. Fibroblasts were also found to be highly enriched in the *COL1* gene in the brain from these transcriptomic studies and have been implicated in fibrotic scar development after CNS injury and postulated to increase the chances of developing AD [115–117]. DEGs from all three- pericytes, smooth muscle cells, and fibroblasts—were associated with dysregulated vasoconstriction and compromised blood flow; these results were consistent with the findings of various mouse models as well [26, 37, 63–65, 76, 118–121]. Upregulated DEGs in mural cells were enriched in immune and pro-inflammatory response pathways, such as cytokines, interleukin-17, and inflammation. It was also observed that *APOD*, which encodes for high-density lipoprotein (HDL), was upregulated in these mural cells [65, 112, 122]. We found that in pericytes, the most upregulated transcripts were enriched for growth factor related genes (*FLT1, SMAD3, STAT3*).

In studies that linked AD GWAS variants [63–65] to single-cell-derived signatures of vascular cells, *APOE*, which has been linked to microglia and astrocytes in AD, exhibited a robust expression in smooth muscle cells. GWAS genes [123–126] such as *ABCA1*, *FHL2*, *HESX1*, and *IL34* were also enriched in fibroblast. *APOE*, which is linked to myeloid cells and astrocytes, was also robustly expressed in human SMCs and meningeal fibroblasts. Interestingly, *PLCG2*, an AD risk gene predominantly expressed in microglia is upregulated in both pericyte and perivascular fibroblast clusters in AD [33, 63]. This provides evidence for an immune-vascular axis in facilitating brain aging and contributing to the genetic risk of AD [127–130].

Overall, these studies highlight the selective vulnerability of mural cells and fibroblasts in AD, characterized by downregulated genes associated with synaptic transmission and cytoskeleton remodeling, and the association to pathways involving fibrotic scarring post-CNS injury. Dysregulated vasoconstriction, compromised blood flow, and upregulated immune response pathways in mural cells are consistent features in AD pathogenesis, with genetic variants like *APOE* robustly expressed in smooth muscle cells and meningeal fibroblasts, indicating an immune-vascular axis in AD. Table 1 compiles some of the most relevant significant DEGS and pathways from all the studies reviewed in this paper, highlighting those linked to vascular cells and associated with neurovascular dysfunction in AD.

**Table 1:** Biological pathways and transcriptomic profiles associated with Alzheimer’s disease in the vascular cells of the NVU and GVU

**Vascular cell types**		**Biological process**	**Cellular pathways**	AD-associated genes (↑ or ↓ indicates up or down-regulation in AD, if applicable)	Refs
Endothelial cells		Angiogenesis	Vascular homoeostasis	CLDN5 ↑, ERG ↑, FLT1 ↑, VWF ↑, ANGPT2 ↑, INSR ↑, ETS1 ↑ ENG ↑, TGM2 ↑, STAT3 ↑, HIF1A ↑, FGF2 ↑ VEGFA ↓, VEGFR2↓, EGFR ↓, SPRED2↓	[33, 62–65, 82–85, 139]
			cell proliferation and endothelial damage	EBF1↓, TSHZ2 ↑, VWF ↑, ABCG2 ↑, ABCB1 ↑ PGRMC1↓	[99, 100]
		Immune response	inflammation	HLA-E ↑, IL6 ↑, IL6R ↑, CR1↓, PLCG2↓, TEK ↓,	[63, 65]
			antigen presenting machinery	B2M ↑, HLA-E↑	[82]
			interferon-γ signaling	IFNγ ↑	[63, 64]
		COVID-19-response	SARS-CoV-2 docking receptors	NRP1↑, BSG↑	[167]
			SARS-CoV-2 host factors	BSG ↑, FURIN↑	[167]
			antiviral defense genes	LY6E ↑, IFITM2↑, IFITM3↑, IFNAR1 ↑	[167]
		BBB integrity	BBB integrity	CTNNB1↑	[94]
			amyloid-beta clearance (p-glycoprotein)	SCARB1 ↑, ATP10A ↓, ABCB1 ↓, PICALM ↓	[65, 94, 99, 139]
		Synaptic Signaling/ ECM Organization	protein folding, cytoskeletal remodeling and intracellular trafficking	HSP90AA1↑, HSPH1↑, HSPA1A ↑, JSPB1 ↑, COL4A1 ↑, INPP5D ↑, CD2AP ↑, ARL15↑, MYRIP ↓, PICALM ↓	[94, 63–65, 139]
Mural cells and fibroblasts	Pericytes	Angiogenesis/ BBB integrity	Dysregulated blood flow, injury & oxidative stress, inflammation,	PLCG2↑, BACH1↑, SLC12A7 ↑, SLC6A12 ↑, SLC19A1 ↑, COL4A1 ↑, CDH6 ↑, SNTB1↑, RIPK2↑, PDGFRβ ↑, EGFR ↓, MEF2C ↓	[33, 62–65, 139]
		Synaptic Signaling/ ECM remodeling	synaptic transmission, cytoskeleton remodeling and contraction, growth factor	FLT1 ↑, SMAD3 ↑, STAT3 ↑, GRM8 ↑ ADAMTS1 ↑, ADAMTS4 ↑, ADAM10, FERMT2 ↑, AGRN ↑, TAGLN ↑ SLC6A1 ↓, MYO1B ↓, DMD ↓	[33, 63–65, 139]
		Cholesterol regulation	HDL metabolism	APOD ↑, SCARB1 ↑	[65]
	Fibroblasts	CNS Injury repair/ ECM remodeling	fibrotic scars formation, structural integrity	COL1 ↑ SPTBN1↓, LAMC1↓	[65, 139]
		Immune Response/ Cell Signaling	Transcriptional regulation, HDL metabolism, inflammation	TAGLN ↑, ABCA1↑, FHL2, ↑, HESX1↑, IL34 ↑, PLCG2↑, APOE ↑, ABCA9 ↑, CEMIP ↑, C7 ↑ SPRED2↓, DAB2IP ↓, DTX2↓, BCL2L1↓, FBLN1	[62–65, 139]
	SMCs	ECM-organization/ Cholesterol metabolism	Inflammation, cytoskeletal remodeling, amyloid-beta clearance,	APOE ↑, ADAMTS1 ↑, ADAMTS4 ↑, FERMT2 ↑, PFDN1 ↓	[63, 65]

*Abbreviations*: *NVU *Neurovascular unit, *GVU *gliovascular unit, *BBB *Blood–brain barrier, *CNS *Central Nervous System, *SMC *Smooth Muscle Cells, *HDL *High Density Lipoprotein

## Cellular crosstalk: interactions of vascular cells with the neuroglial units and astrocytes

The interactions between endothelial cells, mural cells, and fibroblasts are critical to maintaining the structural integrity of the BBB to ensure the regulation of angiogenesis, vascular remodeling, and vessel development [131, 132]. Additionally, the breakdown of these pathways has devastating consequences for neurodegenerative conditions such as AD and has been studied in-depth in murine mouse models. However, limited transcriptomic studies in AD have explicitly looked at vascular communication with microglia, neurons, and astrocytes in the neurovascular unit at a single-cell resolution. A very recent study that built a high-confidence cell-to-cell communication network to investigate signaling differences between AD and healthy brains showed that in pathological states, non-neuronal cells (such as endothelial cells, mural cells, and fibroblasts) and inhibitory neurons globally decrease their connection with most cell types [133]. Therefore, it is important to understand the dysregulated vascular-microglial and vascular-astroglia perturbations from single cell perspectives in human brain samples.

Astrocyte-vascular cell signaling plays a crucial role in coordinating repair and recovery within the neurovascular unit after CNS injury. Studies have proposed that reactive astrocytes act as central organizers of these interactions by engaging in bidirectional communication with vascular cells, influencing angiogenesis, regulating inflammatory responses, and modulating blood–brain barrier integrity. There is also evidence that dynamic activity between endothelial cells and pericytes reciprocally shape astrocyte function, promoting tissue repair and neural recovery in the injured CNS under these circumstances [134, 135]. Furthermore, it has been shown that astrocyte-vasculature interaction mediates nutrient transport into the brain and clearance of brain waste such as potassium ions, amyloid plaques, and phosphorylated tau proteins. This signaling has been deemed crucial to limit inflammatory pathophysiology in the brain and peripheral immune cells [136, 137]. More recently, NicheNet intercellular communication analysis [138] studies identified potential BEC DEG regulators associated with pro-inflammatory and anti-angiogenic gene expression in astrocytes and perivascular macrophages. The authors of this study also showed that risk genes enriched for expression in BEC overlapped substantially with those in microglia and suggested a potential joint contribution of BEC and microglia to the genesis of AD [139].

Other studies have also found similar results showing that the signaling between microglia and vascular cells may influence neurovascular function in AD, as secreted chemokines from activated microglia may regulate endothelial tight junctions integrity and influence the inflammatory state of endothelial walls [140]. The brain regions that express the most variable astrocyte genes are enriched for pathways regulating synaptic transmission, immune cell chemotaxis, and vascular cell proliferation [141].

**Fig. 3:**
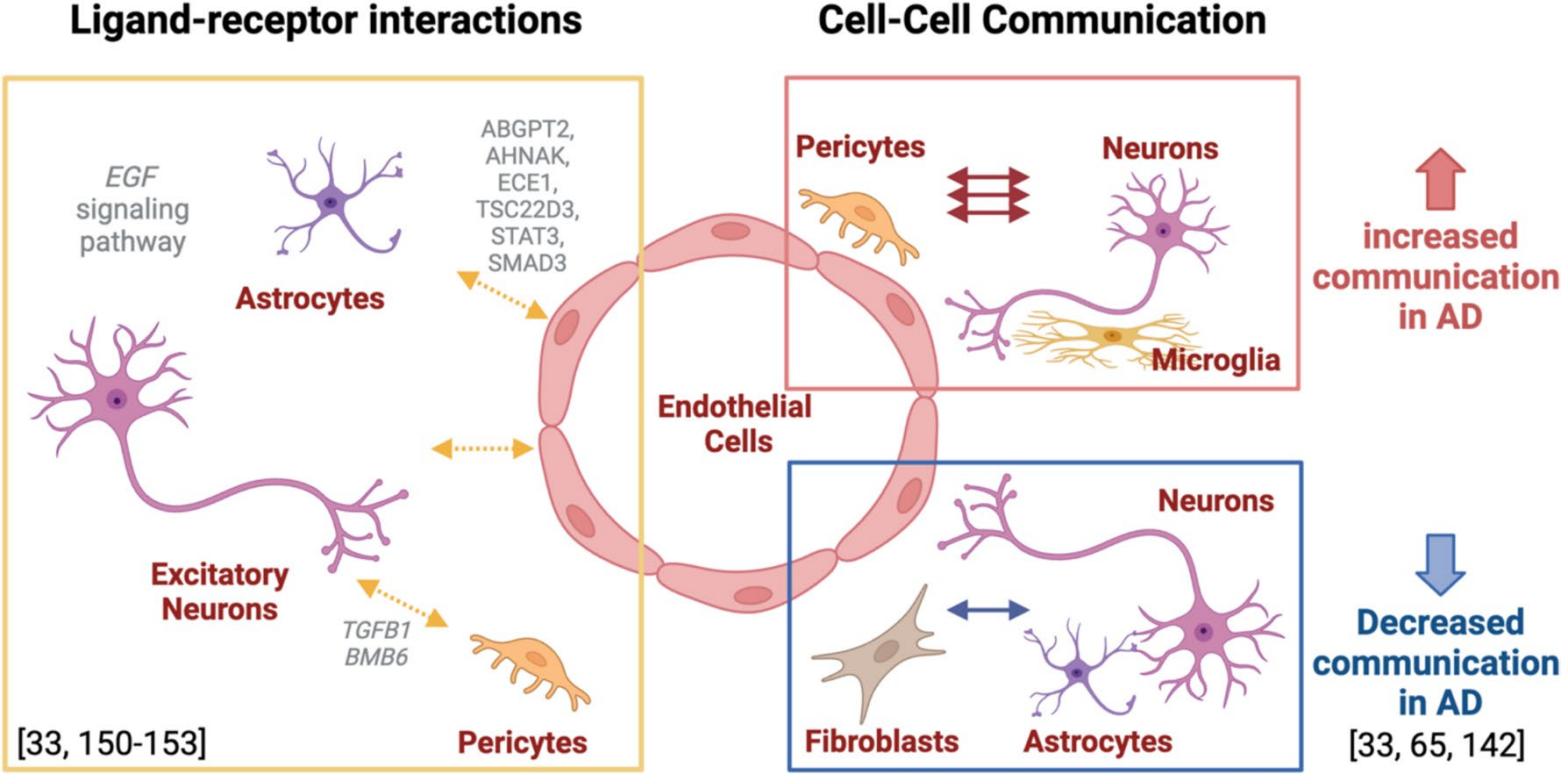
Cellular crosstalk is altered at the NVU and GVU during AD. Evidence of impaired ligand-receptor communications between astrocytes and endothelial as well as neurons and pericytes in AD (left). Pericytes and endothelial cells collectively show increased communication with the neuroglial unit, whereas fibroblast clusters show significantly decreased communication with astrocytes and neurons in AD. Figure created with BioRender.com. Abbreviations: AD, Alzheimer’s disease; NVU, Neurovascular unit; GVU: Gliovascular unit

Some of the recent studies described above, which leveraged in-silico sorting power to isolate and enrich vascular cells, also used computational pipelines focused on the ligand-target relationship to show evidence of an increased communication between pericytes and capillary endothelial cell clusters to the neuroglial unit in AD (Fig. 3). Conversely, the connections between astrocytes and neurons with capillary endothelial cells and fibroblasts were found to be reduced in AD patients (Fig. 3). Furthermore, results from these ligand-receptor and differential cell–cell communication analyses [65, 142] showed that bidirectional cell–cell interaction changes in AD for each pair of cell type identified were significant (*p* < 0.01), identifying a multicellular interaction scheme for the dysregulation of genes in AD across all vascular, astrocytic, glial, and neuronal cell types. This is aligned with results from our study that investigated cell–cell communication within the gliovascular unit amongst endothelial cells, pericytes and astrocytes, where we found AD-related transcriptional changes in astrocytic ligands predicted to bind targets on pericytes and that the top prioritized molecular pair, pericytic *SMAD3* and astrocytic *VEGFA,* influences pericyte functioning as it pertains to BBB regulation [33]. In this study, we also identified vascular-astrocytic molecular interactions in human brains and selected six predicted vascular target genes (*ABGPT2, AHNAK, ECE1, TSC22D3, STAT3, SMAD3*) that had differential expression in AD vs control as well as positive associations with multiple AD endophenotypes [33]. Interestingly, all of these six predicted vascular target genes have been studied in the context of AD-related pathophysiology [133, 143–149].

Collectively, these studies have identified that the largest number of ligand-receptor interactions was shown by four specific cell networks: 1) capillary endothelial cells and astrocytes, 2) capillary endothelial cells and excitatory neurons, 3) pericytes and excitatory cells, 4) pericytes and astrocytes. Additionally, it was shown that TGFB1 exacerbates BBB permeability and regulates pericyte inflammatory response mediated communication with excitatory neurons. BMB6, implicated in dysregulated neurogenesis in AD, was found to mediate increased AD-specific interactions between pericyte subtypes and excitatory neurons, whereas EGF (epidermal growth factor) signaling pathway mediated decreased AD-specific interactions between astrocytes and capillary endothelial cells. However, while not definitively validated, this neurovascular communication between astrocytes and endothelial cells and pericytes is also being studied in multiple animal models as they pertain to critical aspects of AD neuropathology and dysfunction [150–153].

## COVID-19 and Alzheimer’s disease: shared transcriptional signatures and the potential for single-cell studies

Individuals with pre-existing dementia, including AD, are at higher risk of contracting COVID-19, with elevated mortality rates and post-infection cognitive impairments [154–158]. Notably, COVID-19 survivors are at increased risk for new-onset dementia compared to non-infected individuals, emphasizing the need to explore the shared molecular mechanisms underlying both conditions [157, 159]. While multi-omics approaches have provided insight into the overlapping pathways of AD and COVID-19, single-cell studies are limited. Both diseases show alterations in key gene expression patterns, particularly those involved in immune responses and synaptic dysfunction [160–162]. Gene expression studies in COVID-AD patients have shown evidence of increased Aβ burden, disrupted microglial homeostasis, reduced astrocyte population, and dysregulation of oligodendrocyte and myelination pathways [162]. Studies have indicated that peripheral immune dysregulation and pro-inflammatory molecules elevated in COVID-19 can compromise the blood–brain barrier, interacting with malfunctioning astrocytes and microglia implicated in AD pathology, thereby exacerbating neurological damage [163, 164].

Shared signaling pathways, such as PI3K-AKT, Neurotrophin, Rap1, Ras, and JAK–STAT, have been identified through miRNA target predictions, implicating their roles in both AD and COVID-19 [163]. In addition, differentially expressed genes (DEGs) common to AD, COVID-19 patients, and SARS-CoV-2-infected cells have been found to be significantly involved in immune responses, particularly in the modulation of cytokine storms [165, 166]. Network analyses of these DEGs have highlighted the involvement of key immune regulatory genes, including *IRF7*, *STAT1*, *STAT2*, and *OAS1*, which are central to antiviral and immune signaling pathways. Recent single-cell transcriptomic studies have revealed alterations in these genes within brain endothelial cells (BECs), suggesting a pivotal role in neurovascular dysfunction. This study also found *ACE2*, the receptor used by SARS-CoV-2 for cellular entry, was positively correlated with *IRF7* expression in both AD and COVID-19 patients, particularly in neurons and endothelial cells, as demonstrated by snRNA-seq. The authors hypothesized that increased *ACE2* and *IRF7* expression may facilitate greater viral entry and contribute to heightened neuroinflammation and disease progression in AD. Another single cell study [167] comparing the snRNA-Seq profiles of COVID-19-infected AD patients and COVID-19-infected cognitively normal individuals found that two SARS-CoV-2 docking receptors, *NRP1* and *BSG,* had elevated expression in endothelial cells in the prefrontal cortex of both AD patients and healthy controls compared to other brain cell types. The BECs of AD patients and the controls had elevated expression of SARS-CoV-2 host factors (BSG and FURIN) and antiviral defense genes (*LY6E*, *IFITM2*, *IFITM3*, and *IFNAR1*) compared to neurons and other cell types. The authors attributed this to a possible role of brain microvascular injury in COVID-19-mediated cognitive impairment. Additionally, this study also found that individuals with the AD risk allele *APOE ε4/ε4* had reduced transcriptomic expression of antiviral genes compared to *APOE ε3/ε3* AD patients, suggesting that AD patients with the *APOE ε4/ε4* genotype may have less active antiviral defense gene expression activities, rendering them more susceptible to SARS-CoV-2 infection. These findings align with multiple studies suggesting an infection-triggered immune axis as a potential origin of cognitive impairment in AD [168–173].

Neuroimmune dysfunction appears to be a key pathological feature common to both AD and COVID-19, compounding neurodegenerative outcomes. Vascular damage, a frequent consequence of systemic inflammation in COVID-19, is also a critical factor in AD pathogenesis, which is characterized by blood–brain barrier breakdown and vascular inflammation. Applying scRNA-seq to vascular cells, such as endothelial cells and pericytes, could elucidate how SARS-CoV-2 alters gene expression in these populations, particularly genes related to ACE2, inflammation, and oxidative stress, thus deepening our understanding of neurovascular damage in both conditions. Such approaches may also uncover cell-type-specific signaling interactions between vascular cells, neurons, and glia, offering insights into the progression of neurodegeneration in co-affected patients.

Neuroimmune dysfunction has emerged as a central feature in the pathology of both AD and COVID-19, with evidence suggesting that these interactions exacerbate neurodegenerative processes, especially through vascular damage. Given that systemic inflammation in COVID-19 frequently induces vascular injury, and that vascular pathology is a hallmark of AD, exploring these shared pathways is crucial for understanding disease progression. To this end, scRNA-seq holds significant promise in uncovering specific gene expression changes within distinct cell types that might drive neurovascular dysfunction in these conditions. For example– scRNA-seq could differentiate the extent to which ACE2 and other viral receptors like NRP1 and BSG are expressed across diverse vascular and glial cell types in the NVU. This could help clarify which cell types are most susceptible to SARS-CoV-2 infection and whether this susceptibility correlates with increased neuroinflammation and AD pathology. Genes associated with inflammation, such as *TNF-α*, *IL-6*, and *IFN-γ*, may show increased expression in BECs of COVID-19-infected AD patients, indicating that SARS-CoV-2-induced immune responses could accelerate vascular breakdown, further contributing to cognitive decline in AD.

Ligand-receptor pairing analysis across vascular, glial, and neuronal cells could provide insight into how specific cell types communicate during neurodegeneration. In particular, scRNA-seq could be used to map cytokine signaling networks, revealing which cell types are the major producers of pro-inflammatory signals, like *IL-1β* and *IL-6,* and which cells are the primary responders. This could further clarify the mechanisms by which systemic inflammation in COVID-19 accelerates AD pathology, and how certain populations of cells, such as APOE ε4/ε4 carriers, may exhibit differential responses, potentially due to altered receptor expression or impaired immune responses. Continued transcriptomic studies targeting the neurovascular unit (NVU) will be crucial in elucidating the molecular underpinnings of AD and COVID-19, laying the groundwork for more targeted therapeutic strategies.

## Vascular cells for therapeutic targeting in AD: need for a multi-omics approach

**Fig. 4:**
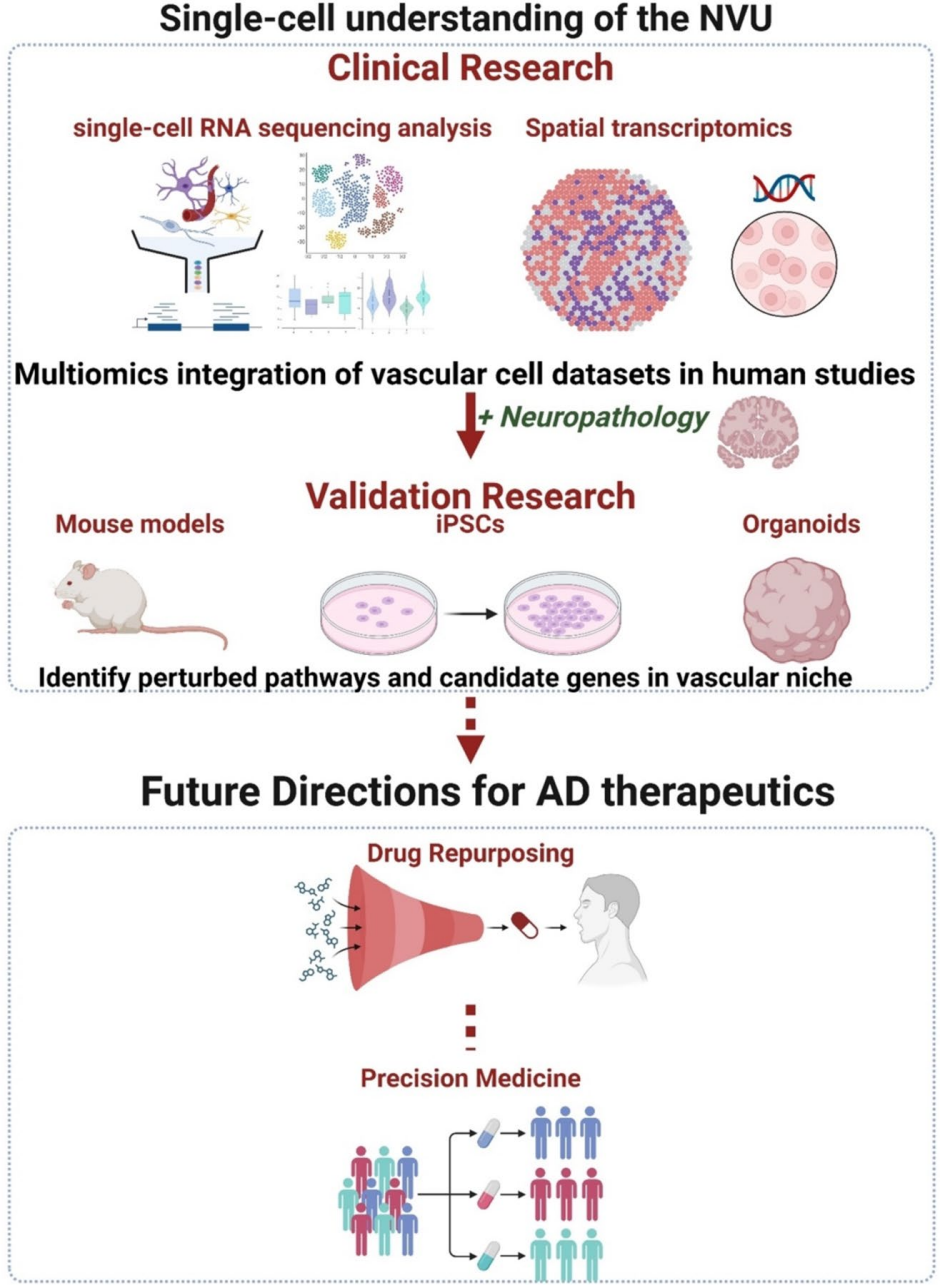
Schematic of understanding perturbed biological pathways in AD using single cell and spatial transcriptomics and its therapeutic implications for precision medicine approaches. Figure created with BioRender.com. Abbreviations: iPSCs, Induced pluripotent stem cells

The findings from rapidly developing single-cell technologies have already provided clues to address the complex mechanisms behind AD pathogenesis (Fig. 4). These results, especially highlighting the cerebrovascular cells, provide ideas for crafting new in-vitro models to understand the brain vasculature in aging and disease and to devise cell-based therapies to mitigate neurovascular dysfunction in AD. However, as we need more robust, integrated, and multifaceted methods to tackle AD, we propose that leveraging single-cell transcriptomics as well as spatial transcriptomics alongside established neuroimaging approaches has emerging potential for elucidating the molecular insights into AD by furthering structural, functional, cellular, and spatial information. Although spatial transcriptomics studies on the NVU and GVU are limited, region-specific profiling of vascular cell subtypes can identify regional depletion patterns and map gene expression of vascular-associated microglia, macrophages, and pericytes, whose interactions with endothelial cells may directly influence neurovascular integrity and function in AD. There is a promising direction for these integrated methods in mapping vascular-specific changes related to key signaling pathways associated with neuroinflammation, synaptic transmission, and BBB integrity. Coupling spatial transcriptomics with single-nucleus RNA sequencing (snRNA-seq) targeted at the NVU allows for elucidating how these pathways are disrupted across vascular cells, microglia, and astrocytes, and understanding their contributions to the dysregulation of cerebrovascular dynamics observed in AD.

Additionally, incorporating neuroimaging data from established methodologies such as MRI and PET scans may be used to correlate structural and functional alterations in the cerebral vasculature with molecular changes observed in vascular cell subtypes—providing a huge step towards understanding vascular cell-specific underpinnings of brain-level AD changes. Analyzing neuroimaging biomarkers such as cerebral blood flow alterations, blood–brain barrier integrity, and amyloid deposition concurrently with single-cell and spatial transcriptomic data from vascular cells can indicate how cerebrovascular pathology presents itself at the cellular level in AD. Currently, several neuroimaging techniques [174–180] are used to detect vascular alterations in AD (MRI, PET, CT) such as vascular calcifications in the hippocampus, BBB leakage, microbleeds, and white-matter hyperintensity (WMH); however, these techniques should be supplemented with brain-level single-cell and spatial transcriptomic data to form a comprehensive map of vascular alterations in AD that encompass intracellular as well as intercellular connections of the NVU components and their subsequent dysregulation. The emerging landscape of ADRD fluid biomarkers offers a minimally invasive means of validating the NVU specific molecular signatures associated with neurodegeneration and vascular injury in such single cell and spatial transcriptomic studies. For instance, elevated plasma GFAP levels, a marker for reactive astrocytes, have been associated with Aβ pathology, reflecting glial responses to AD. These biomarker findings align with single-cell revealed molecular features of astrocytes and endothelial cells, which suggest that dysregulation of gliovascular cells and the NVU/GVU are key contributors to neurodegenerative processes. Our recent findings [33] show that blood transcript levels of *SMAD3*, a pericytic molecule that is perturbed in AD brains and essential in BBB function, also associate with neuroimaging phenotypes of amyloid PET and brain cortical thickness, provide proof of principle support for the significant potential of combined multi-omics and deep phenotypic data in discovering novel AD biomarkers. Such multi-modal data and novel analytic approaches can pave the way for centrally linked peripheral biomarkers that can inform on brain molecular perturbations in AD that can be detected in blood (see also clear-ad.org). Future research integrating fluid biomarkers with neuroimaging and high-throughput single-cell transcriptomics will be critical in validating region- and cell-type-specific vascular markers in AD, alongside in vivo evidence of NVU/GVU alterations [181].

Given the advancement in systems biology approaches of multi-omics integration, the landscape of therapeutic target identification using these approaches and pathways identified in vascular cells could elevate our understanding of prioritizing candidate targets based on their association with cerebrovascular dysfunction, amyloid, and cognitive decline in AD. As attempted by some studies [142, 182–187] already, individually as well as by high-throughput integration, single cell and spatial transcriptomics technologies in AD open opportunities for 1) drug repurposing based on identified targets within vascular cell subtypes, 2) creation of biomarkers of vascular dysfunction based on perturbed vascular molecules, and 3) crafting therapeutic targets based on molecular pathways affecting cell–cell communication in the neurovascular unit. By synergizing the capabilities of single-cell transcriptomics, spatial transcriptomics, and various multi-omics technologies, a promising avenue emerges for the development of precision therapeutics in AD. This strategic integration holds significant potential in directing therapeutic interventions towards vascular cells and addressing the pathology associated with AD.

## Conclusion and future perspectives

The main goal of this review was to compile significant single-cell studies that examined the transcriptional alterations of the NVU and GVU components in human brains. Until now, single-cell technologies have provided valuable insights into the cellular heterogeneity of AD; however, several outstanding questions remain regarding the cellular and molecular mechanisms regulating neurogliovascular units, including the contribution of vascular cell subtypes to vascular dysfunction in AD and the potential therapeutic implications of targeting the astrocyte-immune–vascular axis. The findings from these snRNA-seq studies suggest the 1) selective vulnerability of endothelial, mural, and fibroblast cells of the NVU or GVU in AD pathogenesis, 2) its crucial interaction with astroglia, immune cells, and neuronal units, and 3) upregulation of an inflammatory response associated with BBB integrity and neurovascular coupling. Limited by small sample sizes, sequencing strategies, and computational capability, our understanding of the mechanisms behind the coordinated activation of immune-related genes in microglia, astrocytes, and vascular cells during neurodegeneration and brain aging remains incomplete. Additionally, the mechanisms by which perivascular macrophages and other immune cells interact and regulate the function of vascular cells in AD are still not fully understood. Lastly, it remains to be investigated whether the dysfunction of these vascular cells drives the neurodegeneration observed in AD. Continued advancements in sample collection, computational methods, and the integration of techniques are essential for leveraging single-cell transcriptomics to advance our understanding of the neurovascular unit and AD pathogenesis and develop effective therapies. Especially, the integration of single-cell transcriptomics of vascular cell subtypes with spatial transcriptomic technologies presents a powerful approach to unraveling the complex interplay between cerebrovascular dysfunction and AD pathogenesis. The emerging field of single-cell transcriptomics holds a remarkable potential to accelerate our understanding of AD-associated vascular changes that will ultimately contribute to the development of therapeutic targets and robust biomarkers for early diagnosis of AD.

## Supplementary Information

Additional file 1.

## Data Availability

Not applicable.

## Abbreviations

AD: Alzheimer’s disease
ADRD: Alzheimer’s disease and related dementias
snRNA-Seq: Single nuclei RNA sequencing
scRNA-Seq: Single cell RNA sequencing
CAA: Cerebral Amyloid Angiopathy
NVU: Neurovascular unit
CNS: Central Nervous System
BBB: Blood–brain barrier
iPSCs: Induced pluripotent stem cells
MRI: Magnetic Resonance Imaging
PET: Positron Emission Tomography
SMC: Smooth muscle cells
BEC: Brain endothelial cells
ECM: Extracellular matrix
